# Effects of Silver Nanoparticle‐Loaded 18β‐Glycyrrhetinic Acid on P2X7 Receptor and Endoplasmic Reticulum Stress‐Mediated NLRP3 Inflammasome Activation in Testicular Tissue in an Animal Diabetes Model

**DOI:** 10.1002/fsn3.70893

**Published:** 2025-11-21

**Authors:** Volkan Gelen, Ali Yeşildağ, Serkan Ali Akarsu, Hülya Kara, Deniz Tekiner, Adem Kara

**Affiliations:** ^1^ Department of Physiology, Faculty of Veterinary Medicine Kafkas University Kars Turkey; ^2^ Department of Bioengineering, Faculty of Engineering and Architecture Kafkas University Kars Turkey; ^3^ Department of Reproduction and Artificial Insemination, Faculty of Veterinary Medicine Atatürk University Erzurum Turkey; ^4^ Department of Anatomy, Faculty of Veterinary Medicine Atatürk University Erzurum Turkey; ^5^ Department of Histology and Embryology, Faculty of Veterinary Medicine Atatürk University Erzurum Turkey; ^6^ Department of Genetics, Faculty of Science Erzurum Technical University Erzurum Turkey

**Keywords:** 18β‐glycyrrhetinic acid, AgNPs, diabetes, ER‐stress, NLRP, P2X7, rat, testis

## Abstract

Diabetes, a metabolic disease characterized by high blood sugar, causes damage to many organs in the body. Accordingly, the present study investigates the effects of silver nanoparticle‐loaded 18β‐glycyrrhetinic acid (AgNP+18β‐GA) on P2X7 receptor and ER stress‐mediated NLRP3 inflammasome activation in testicular tissue. 42 Wistar‐Albino rats aged 6 months were used. The rats were divided into the following seven groups: Control, AgNP+18β‐GA100, DM, DM‐18β‐GA100, DM‐AgNP+18β‐GA50, DM‐AgNP+18β‐GA100, and DM‐AgNP. After the experiment, sperm samples obtained from the rats were evaluated. Oxidative stress markers, serum IL‐1β, and TNF‐α levels were determined in the testicular tissue samples taken separately. In addition, Caspase‐1, NLRP3, P2X7, Caspase‐3, NF‐κB, IRE1, ATF6, and CHOP protein expression levels were evaluated using Western blot analysis of histopathological examinations. Specifically, the most significant decrease was observed in NLRP3, Caspase‐1, P2X7, and NF‐κB levels in the DM‐AgNP+18β‐GA100 group (*p* < 0.05). About sperm parameters, while sperm motility and viability significantly decreased in diabetic groups (*p* < 0.05), a significant improvement in these values was observed in the AgNP+18β‐GA applied groups (*p* < 0.05). AgNP+18β‐GA improved sperm morphology and histopathological damage in diabetes‐induced testicular damage, as well as inhibited P2X7 receptor and endoplasmic reticulum stress‐mediated NLRP3 inflammasome activation.

## Graphical abstract

Effects of silver nanoparticle‐loaded 18β‐glycyrrhetinic acid on P2X7 receptor and endoplasmic reticulum stress‐mediated NLRP3 inflammasome activation in testicular tissue in an experimental diabetes model in rats.

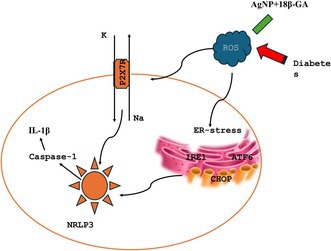

## 1 Introduction

Diabetes mellitus (DM), a globally common metabolic disease characterized by high blood sugar levels, causes significant health problems (Zheng et al. 2018). Specifically, by increasing the risk of micro and macrovascular diseases, diabetes can negatively affect the functions of many organs. DM is defined as the epidemic of the 21st century (Malek et al. 2019). According to a report of the International Diabetes Federation, as of 2021, there were 537 million diabetic patients worldwide, which is steadily increasing. A significant breakthrough in diabetes research was the discovery of the role of insulin in glucose metabolism in the early 1920s (Zaccardi et al. 2016; Banting et al. 1922). Insulin is produced in the β‐cells of the pancreas and controls blood sugar levels by regulating glucose metabolism. This discovery revolutionized the treatment of diabetes and significantly contributed to the understanding of the pathophysiology of the disease. In terms of clinical classification, diabetes is commonly categorized into type 1 and type 2 diabetes (Atkinson et al. 2014). Type 1 diabetes is a relatively rare autoimmune disease that occurs when the immune system attacks the insulin‐producing β‐cells of the pancreas (Katsarou et al. 2017). This disease usually begins in childhood or adolescence and continues throughout life. Individuals with type 1 diabetes must receive continuous insulin therapy due to the loss of insulin production in their pancreas (Daneman 2006; Paschou et al. 2018). By contrast, type 2 diabetes, which is considerably more common, is usually observed in middle‐aged and older individuals and develops in association with insulin resistance (Chatterjee et al. 2017; DeFronzo et al. 2015). Type 2 diabetes accounts for 90% of all cases of diabetes and is usually managed with lifestyle changes, diet, and medication.

Along with worsening blood sugar levels, diabetes can also adversely affect the functions of many organs, including the testicles (Thomas et al. 2015; Khanolkar et al. 2008; Pasquier et al. 2006). The effects of diabetes on the testicles are associated with mechanisms such as vascular damage, oxidative stress, insulin resistance, glucose toxicity, accumulation of glycation end products, inflammation, and apoptosis (Lee and Hasler 2017; Biessels et al. 2006; Umemura et al. 2017). Although the mechanisms of diabetic testicular damage are not fully understood, these processes are thought to have negative effects on reproductive functions and may lead to infertility. The effects of diabetes on the testicles are particularly related to damage to the blood‐testis barrier in the testicles and the resulting deterioration in spermatogenesis. Accordingly, previous research has focused on potential treatment options for diabetic testicular damage. A particular research interest was elicited by 18β‐glycyrrhetinic acid (18β‐GA), which is known for its antioxidant, anti‐inflammatory, and anti‐allergic properties (Shinu et al. 2023; Gupta et al. 2023; Bezerra et al. 2023). For its therapeutic or protective effects in organ toxicity models and the potential to modulate oxidative stress and inflammation in the body, 18β‐GA stands out as a protective agent against damage caused by diabetes (Türkmen et al. 2023; Wu et al. 2021). However, previous research on the protective role of 18β‐GA against diabetic testicular damage remains scarce. In particular, none of the previous studies has investigated the effects of 18β‐GA loaded with silver nanoparticles on testicular tissue.

Silver nanoparticles are a nanotechnological delivery vehicle that can help to target and increase the efficacy of biologically active compounds. Due to their ability to cross the blood‐testis barrier, these nanoparticles are effective in transporting pharmaceutical agents to testicular tissue (Lee and Melsel 1982). The potential of 18β‐GA loaded with silver nanoparticles to reduce diabetic testicular damage may increase the efficacy of this compound and provide a new approach to treatment. By increasing the bioavailability of the compound, silver nanoparticles may play a more effective role in modulating mechanisms associated with oxidative stress, inflammation, and endoplasmic reticulum (ER) stress in testicular tissue. However, it is not yet known whether combining 18β‐GA with AgNPs offers superior protective efficacy compared to using either agent alone. We hypothesize that loading 18β‐GA onto AgNPs will enhance its stability, cellular uptake, and targeted delivery to testicular tissue, thereby producing synergistic effects against diabetes‐induced oxidative stress, inflammation, and ER stress.

In this context, the present study aims to investigate the effects of 18β‐GA loaded with silver nanoparticles on the activation of the P2X7 receptor and ER stress‐mediated NLRP3 inflammasome in testicular tissue in an Alloxan‐induced diabetic rat model. We also explore the mechanisms of the protective effects of 18β‐GA against diabetic testicular damage. Oxidative stress, inflammation, and ER stress‐related protein expressions in testicular tissue are evaluated using Western blot analysis. The results on the potential therapeutic role of 18β‐GA loaded with silver nanoparticles and its protective effects against diabetic testicular damage contribute to the development of new treatment strategies to alleviate the damage caused by diabetes.

## 2 Materials and Methods

### 2.1 Synthesis and Characterization of Silver Nanoparticles (AgNP) and Silver Nanoparticles/18β‐Glycyrrhetinic Acid (AgNP/18β‐GA)

AgNPs were synthesized using a chemical reduction method that reduces the silver ion (Ag^+^) in AgNO_3_ to silver nanoparticles; citrate was used as the reducing and stabilizing agent (Ahmed et al. 2016). To synthesize AgNP, 0.0167 g silver nitrate (AgNO_3_) was dissolved in 100 mL of distilled water. Then, 20 mL of distilled water was added to a flask containing 0.020 g of sodium citrate (Na_3_C_6_H_5_O_7_). AgNO_3_ was heated to boiling point using a magnetic stirrer. This was followed by adding 5 mL of the prepared solution (Na_3_C_6_H_5_O_7_) dropwise to the boiling AgNO_3_ solution. The mixture was then heated on a magnetic stirrer for about 1 h or until the color of the mixture changed from yellow to brown. The characterization of AgNP and AgNP/18β‐GA was first performed using ultraviolet–visible spectroscopy (UV–Vis) (Multiskan Sky, Thermo Scientific, Waltham, MA, USA) operating in the wavelength range from 200 to 900 nm. In addition, Fourier transform infrared spectroscopy (FTIR) with a PerkinElmer‐Tensor 27 (Bruker, Billerica, MA, USA) operating in the wavelength range of 400–4000 cm^−1^ was used for the characterization of AgNP and AgNP/18β‐GA structures. A Hitachi model HighTech‐7700 (Tokyo, Japan) was employed as a transmission electron microscope (TEM). Scanning electron microscopy (SEM) data analyses were performed with a FEI QUANTA model FEG 450 Field Emission Scanning Electron Microscope (FEI Technologies Inc., Hillsboro, OR, USA).

### 2.2 Formation of Experimental Diabetes Model, Preparation of Chemicals, and Implementation of Applications

The study was approved by Kafkas University Animal Experiments Local Ethics Committee (KAÜ‐HADYEK/2023‐036). All experiments were performed in accordance with the European Directive 2010/63/EU for animal experiments. In addition, all animal‐related procedures used in this study were performed in accordance with Animal Research: Reporting of In Vivo Experiments (ARRIVE) guidelines. Saline was administered to the control group by a single intraperitoneal injection. DM, DM‐18β‐GA100, DM‐AgNP+18β‐GA50, and DM‐AgNP+18β‐GA100 groups were administered a single intraperitoneal injection of alloxan monohydrate (Sigma Co., USA) at a dose of 120 mg/kg body weight dissolved in normal saline to create experimental diabetes. To confirm diabetes, blood glucose concentrations were measured using blood samples taken from the tail vein. Blood glucose measurements were performed using a glucometer and test strips [Accu‐Check Active] at the first hour (before the start of the experiment) and then at 48 h, 1 week, and the end of the experiment. Blood glucose levels above 220 mg/dL were considered diabetic and were included in the study. Then, 18β‐GA100, DM‐AgNP18β‐GA50, and DM‐AgNP18β‐GA100 (Chu et al. 2021) groups were administered the indicated doses via gavage for 21 days (see Table 1).

**TABLE 1:** Experimental groups and applications.

Group name	Number of animals	Applications
Control	6	ip saline
AgNP+18β‐GA100	6	AgNP18β‐GA100
DM	6	Alloxan 120 mg ip
DM‐18β‐GA100	6	Alloxan 120 mg ip +18β‐GA100
DM‐AgNP+18β‐GA50	6	Alloxan 120 mg ip + AgNP18β‐GA50
DM‐AgNP+18β‐GA100	6	Alloxan 120 mg ip + AgNP18β‐GA100
DM‐AgNP	6	Alloxan 120 mg ip + AgNP

### 2.3 Termination of the Study

At the end of the treatment period, all rats in the experimental groups were euthanized under Xylazine (4 mg/kg, ip) and ketamine (40 mg/kg, ip) anesthesia after 12 h of overnight fasting. Blood samples were taken intracardially, and the testicular tissues were removed. Some of the testicular tissues were fixed by the immersion method in 10% buffered formaldehyde solution for the subsequent histopathological analysis, while the remaining tissues were stored in a −80°C deep freezer until biochemical and Western blot analyses were performed.

### 2.4 Histological Evaluation of the Testis

Testicular tissue samples taken from rats were fixed in 10% buffered formaldehyde solution for 24–48 h. After the fixation process was completed, the tissues were cleaned by washing with tap water for 24 h. Then, the samples were passed through graded alcohol and xylene series, respectively, and embedded in paraffin blocks. 5 μm‐thick sections were taken from the obtained paraffin blocks using a Leica RM2125RT microtome (Leica Microsystems, Wetzlar, Germany). For histopathological evaluation, sections from all groups were stained with Mallory's triple staining technique modified by Crossman. Microscopic examination of the sections was performed using a Zeiss AXIO Scope A1 (Germany) model camera‐attached microscope. Histological damage and spermatogenesis status observed in testicular sections were examined using Johnsen's mean testicular biopsy score. This examination was based on the presence or absence of germ cell types in the testicular seminiferous tubules. For each testicular sample, 30 tubules were evaluated for spermatozoa, spermatids, spermatocytes, spermatocytes, spermatogonia, germ cells, and Sertoli cells. On the basis of the results of this evaluation, each tubule was ranked from 1 to 10. A high Johnsen score represents healthy spermatogenesis status, while a low score indicates a more pronounced dysfunction. Further detail on the scoring approach is provided in Table 2.

**TABLE 2:** Histological classification of seminiferous tubular sections according to the Johnsen scoring system.

Score	Definition
10	Complete spermatogenesis is observed, with many spermatozoa; the germinal epithelium has a regular thickness
9	Many spermatozoa are present, but the germinal epithelium appears irregular
8	Only a few spermatozoa are present in the section
7	No spermatozoa, but many spermatids
6	No spermatozoa and only a few spermatids
5	No spermatozoa or spermatids, but a few or many spermatocytes
4	Only a few spermatocyte cells are present; no spermatids or spermatozoa
3	Only spermatogonium cells are present, other germ cells are absent
2	No germ cells are present, but Sertoli cells are visible
1	No germ cells or Sertoli cells are present in the tubular section

### 2.5 Evaluation of Sperm Samples Obtained From Rats and Biochemical Analysis of Testicular Tissue

After the experiment was terminated, superoxide dismutase (SOD) activity, malondialdehyde levels (MDA), and glutathione (GSH) levels in the testicular tissues of rats in each group were analyzed using ELISA commercial kits. At the end of the experiment, rats were euthanized with sevoflurane (SevoFlo, Abbot Laboratories) inhalation anesthesia. The testes were removed, the cauda epididymis was cut and separated from the testes, and the connective tissues were cleaned using anatomical scissors. The testes were then weighed. The right cauda epididymis was used to obtain sperm, while the cauda epididymis was minced with anatomical scissors, and the semen sample was taken into 5 mL physiological water in a Petri dish. The cauda epididymis was kept at room temperature for 10 min for spermatozoa passage from the epididymal tissue to the liquid. The connective tissues were cleaned using anatomical scissors, and the obtained liquid was used as the “semen sample”. The right cauda epididymis was used for motility and other sperm analyses, while the left cauda epididymis was used for density determination by the retrograde flushing method.

For each animal, semen was obtained from the right cauda epididymis in a Petri dish by the retrograde flushing method. The obtained liquid was used for motility determination. A phase contrast microscope (Primo Star; Zeiss, Germany) with a heating table (35°C) was used to evaluate motility in epididymal sperm. The semen sample (20 μL) was placed on the slide and covered with a coverslip. Motility percentage was then evaluated subjectively. Sperm motility estimates were made in three different areas on the slide for each sample, and the average value of these areas was determined as the “motility score”.

Cauda epididymis sperm density was determined by the retrograde flushing technique using a modification of the method as previously described by Akarsu et al. (2024b). After the cauda epididymis was excised, it was placed in 35 mm petri dishes containing 5 mL of Tris solution. The cauda epididymis was then sectioned from several places and incubated at 37°C. The sperm suspension was slowly drawn into transfer pipettes. After centrifugation to obtain pure sperm, 5 μL of the obtained sperm were taken and mixed with 995 μL of distilled water in an Eppendorf tube. The obtained mixture was vortexed to make it homogeneous. Then, the number of spermatozoa was counted with a Thoma slide. The abnormal sperm cell rate was evaluated with the method previously proposed by Akarsu et al. (2024a). Briefly, an equal amount of eosin‐nigrosin dye (1.67% eosin, 10% nigrosin, and 0.1 M sodium citrate) was placed on 20 μL of the semen sample and mixed. A smear was taken from the obtained mixture with the help of a coverslip and left to dry. The preparations were then evaluated under a light microscope at 400× magnification. A total of 300 spermatozoa were examined in each sample, and the total of acrosome (wide, granular, curved, small, acrosomeless, degenerate), head (narrow, pear‐shaped, spear‐shaped, shovel‐shaped, bulb‐shaped), body (short, wide, thin, deformed, broken, folded, axial type, fibrillar) and tail section anomalous cells (proximal and distal protoplasmic droplet, knotted tail, coiled tail, rudimentary tail, fibrillar tail, double‐headed tail) were expressed as percentages. Sperm DNA fragmentation was determined by acridine orange (AO) staining method using a fluorescent microscope (Zeiss Axioscope, Germany). 20 μL of semen were placed on the slide and a smear was taken. First, it was air‐dried and then fixed overnight in the freshly prepared Carnoy solution (methanol/acetic acid, 3:1). A mixture of 1% AO in 10 mL of distilled water was added to a mixture of 40 mL of 0.1 mol/L citric acid and 2.5 mL of 0.3 mol/L Na_2_HPO_4_·7H_2_O. The previously prepared AO solution was stored in the dark for 4 weeks, after which it was examined with a fluorescence microscope.

### 2.6 Western Blot Analysis

The protein expression changes in rat brain tissue samples were examined using Western blot analysis. Antibodies with specific reactivity with rats (Table 3) were used in the WB analysis. A “blotting stack” was created for the transfer of the serum proteins to the PVDF membrane. To this end, first, a stack of 8 filter papers was placed at the bottom of the semi‐dry “Turbo blotter” transfer cassette and placed on a PVDF membrane with a pore size of 0.2 μm. Then, the SDS‐PAGE gel was placed on the PVDF membrane, and air bubbles that could have remained between the gel and the membrane or filter papers were removed with the help of a small roller. Finally, a stack of 8 filter papers was placed on the gel, and the lid of the Turbo blotter cassette was closed and placed in the device. The blotting process was performed at 1.3 A, 25 V for 7 min. At the end of the process, the PVDF membrane was incubated with blocking buffer for 1 h, and at the end of the period, a primary antibody at a concentration of 5 μg/mL prepared in blocking buffer was added to the membrane and incubated overnight in the refrigerator on an orbital shaker. When the primary antibody incubation was completed, the membrane was washed with PBS‐T 5 times for 5 min. Then, a secondary antibody at a concentration of 1 μg/mL prepared in the blocking buffer was added and incubated at room temperature for 1 h. At the end of the period, the membrane was washed with PBS‐T 5 times for 5 min. Finally, the chemiluminescent HRP substrate prepared according to the manufacturer's recommendations was added to the membrane and the blots were photographed with a gel imaging system (GE LAS 500) in the chemiluminescent mode. The intensity levels in the blots were determined using the TL 120 program (The Complete Guide to Western blottıng; https://www.ptglab.com/media/1474/wb‐collection_for‐web.pdf). “Total protein normalization” was used for normalization of protein expression levels. To this end, the total protein amount obtained as a result of gel analysis of a sample was divided by the amount of blot on the membrane, and the normalization process was performed.

**TABLE 3:** Primary and secondary antibodies β.

Antibody	Manufacturer	Dilution
Caspase‐3	Santa Cruz, sc‐56,053	1/1000
Caspase‐1	Santa Cruz, sc‐56,036	1/1000
Nf‐kB	Affinity Biotech, AF5103	1/1000
P2X7	Affinity Biotech, sc‐514,962	1/1000
NLRP3	Santa Cruz, sc‐134,306	1/1000
CHOP	Affinity Biotech, BF8018	1/1000
IRE1	Affinity Biotech, DF7709	1/1000
ATF6	Affinity Biotech, DF6009	1/1000
Beta‐Actin	Santa Cruz, sc‐47,778	1/2000
Secondary antibody (Goat anti‐rabbit IgG‐HRP)	Santa Cruz, SC‐2004	1/2000
Secondary antibody (Mouse anti‐rabbit IgG‐HRP)	Santa Cruz, SC‐2357	1/2000

## 3 Results

### 3.1 Characterization of AgNP and AgNP/18β‐GA

**FIGURE 1:**
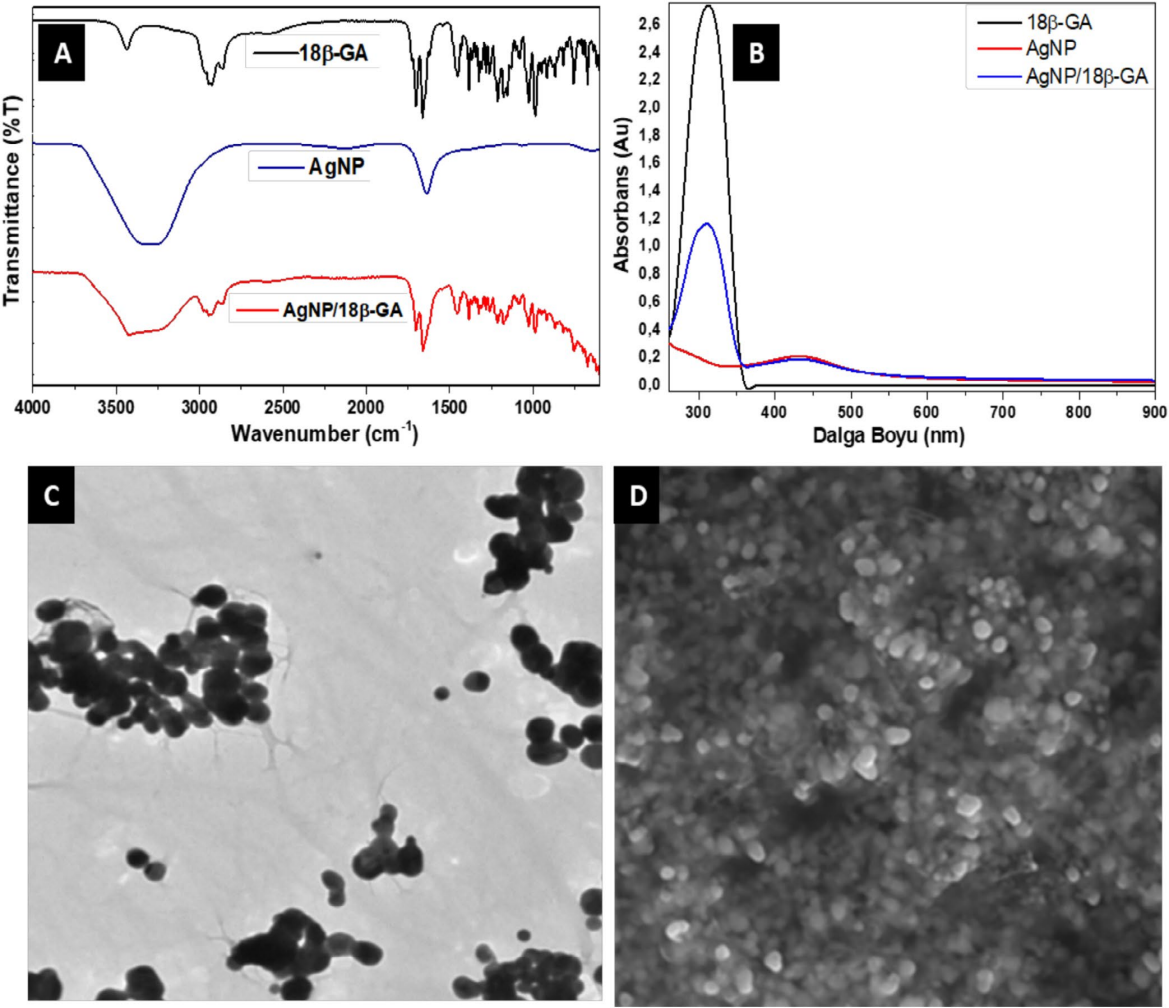
(A) FT‐IR spectrum of 18β‐Glycyrrhetinic acid, AgNP, and AgNP/18β‐GA. (B) UV of 18β‐Glycyrrhetinic acid, AgNP, and AgNP/18β‐GA. (C) The TEM of AgNP. (D) SEM of AgNP.

For the characterization of 18β‐GA, AgNP, and AgNP/18β‐GA, spectroscopic techniques such as FT‐IR, UV, TEM, and SEM were used. First, FT‐IR spectroscopic analysis was performed to characterize the bond linkages and functional groups of the analyzed materials. FT‐IR spectra of 18β‐GA, AgNP, and AgNP/18β‐GA are shown in Figure 1A. Significant differences were observed when the FT‐IR spectra of 18β‐GA, AgNP, and AgNP/18β‐GA were analyzed. These differences primarily indicate the formation of AgNP. Moreover, they also confirm the formation of the AgNP/18β‐GA structure due to the electrostatic interaction between 18β‐GA and AgNP. The bands observed in the FT‐IR analysis indicate the components in the synthesized AgNP and AgNP/18β‐GA. Next, the FT‐IR bands were compared with standard values to identify functional groups. In Figure 1A, the narrow band at approximately 3380 cm^−1^ for 18β‐GA and the broad bands at approximately 3340 cm^−1^ for AgNP and AgNP/18β‐GA indicate the presence of the O‐H group in these compounds. The presence of strong bands at about 1630 cm^−1^ due to ‐CH groups was observed. The peaks at 1700 cm^−1^, 1281 cm^−1^, 1272 cm^−1^ and 772 cm^−1^, 756 cm^−1^, 581 cm^−1^ show stretching and vibrations in C=O groups and C=C groups. These FT‐IR results confirm the formation of AgNP and AgNP/18β‐GA. The chemical reaction indicating AgNP formation is known to cause a visible color change of the reaction mixture from yellow to brown. This color change confirms the formation of AgNPs as a result of the reduction of Ag^+^ ions to Ag^0^. Previous studies have pointed out that the UV absorption peak of AgNP is observed between approximately 400–450 nm and is generally spherical or round in shape. Moreover, it was confirmed by UV–Vis spectra of Surface Plasmon resonance (SPR), which exhibits a strong absorption peak in the UV spectrum of AgNP between about 450–500 nm, a typical band for AgNP as a result of the reduction of Ag ions. In the present study, this absorption peak at approximately 445 nm, indicating the formation of AgNP, was not observed in the UV spectrum of 18β‐GA and was observed in the UV spectrum of both AgNP and AgNP/18β‐GA. This confirmed the formation of AgNP and AgNP/18β‐GA structure (see Figure 1B). TEM spectroscopy was used to determine the shape and size of AgNP. Figure 1C shows the TEM image of the synthesized AgNP. The TEM image showed that AgNPs have a round or spherical morphology, with an average size of approximately 10–100 nm. Figure 1D shows a scanning electron microscopy (SEM) image of the synthesized AgNP. The results of SEM analysis showed that, in addition to the size of the synthesized AgNP, their distribution was also quite well dispersed, and the AgNP particles had different diameters and sizes ranging from about 10 to 150 nm, thus supporting the TEM results (Naganthran et al. 2022).

### 3.2 Blood Glucose Values and HbA1c Levels of the Experimental Groups

**FIGURE 2:**
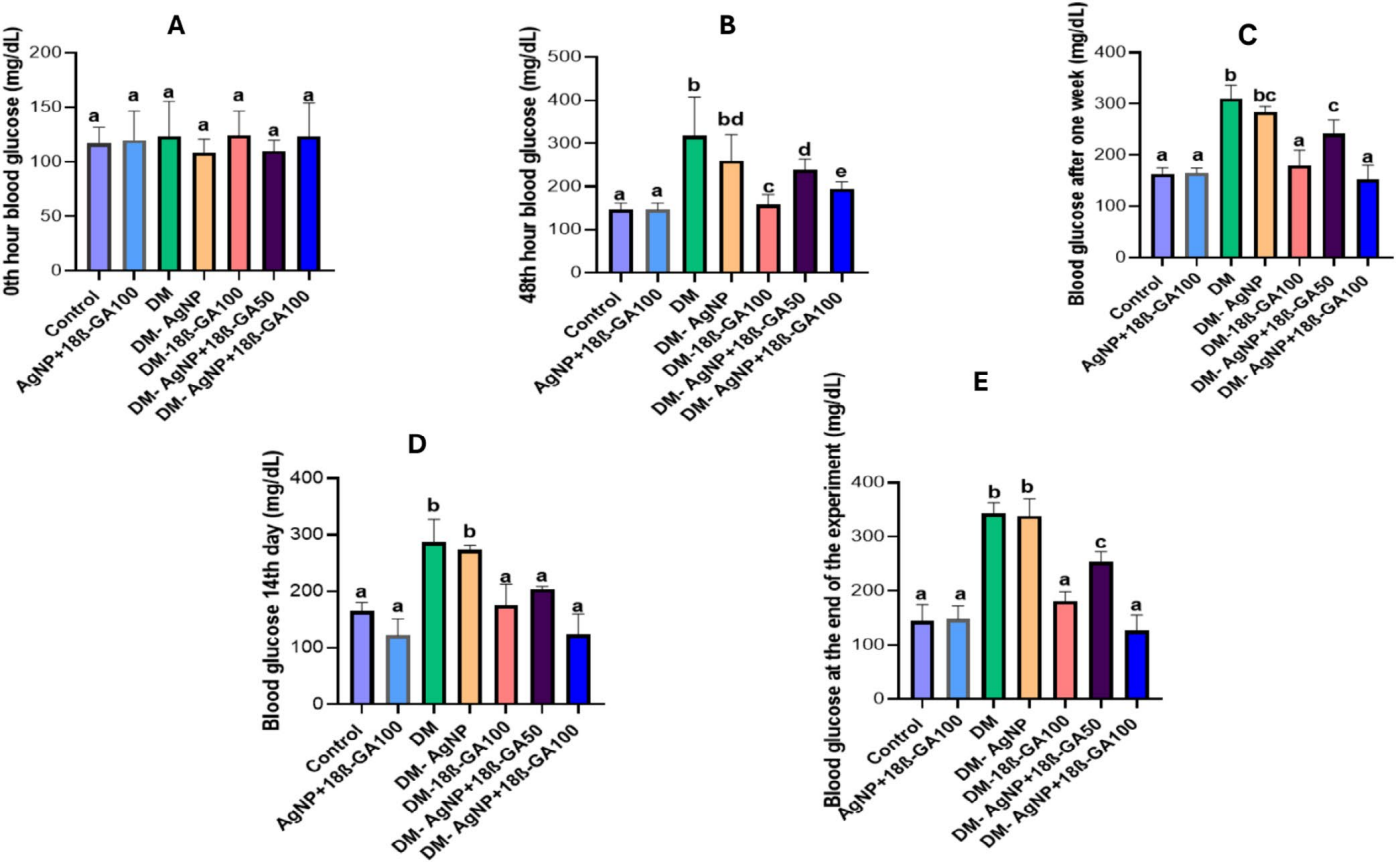
Blood glucose values of blood samples obtained from the experimental groups at the beginning of the experiment, at 48 h, 1 week, on day 14, and at the end of the experiment.

**FIGURE 3:**
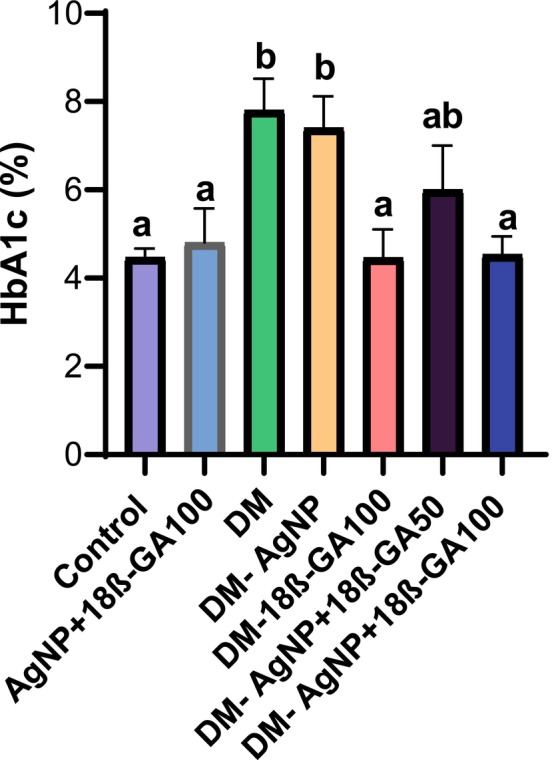
Blood HbA1c percentage values obtained from the experimental groups.

Blood glucose values measured from blood samples obtained from the experimental animals before the experiment (A), 48 h after the experiment (B), 1 week later (C), on day 14 (C), and at the end of the experiment (E) are shown in Figure 2. In addition, serum HbA1c levels obtained from blood samples after the applications were determined. The findings are shown in Figure 3.

### 3.3 Oxidative Stress Parameters

**FIGURE 4:**
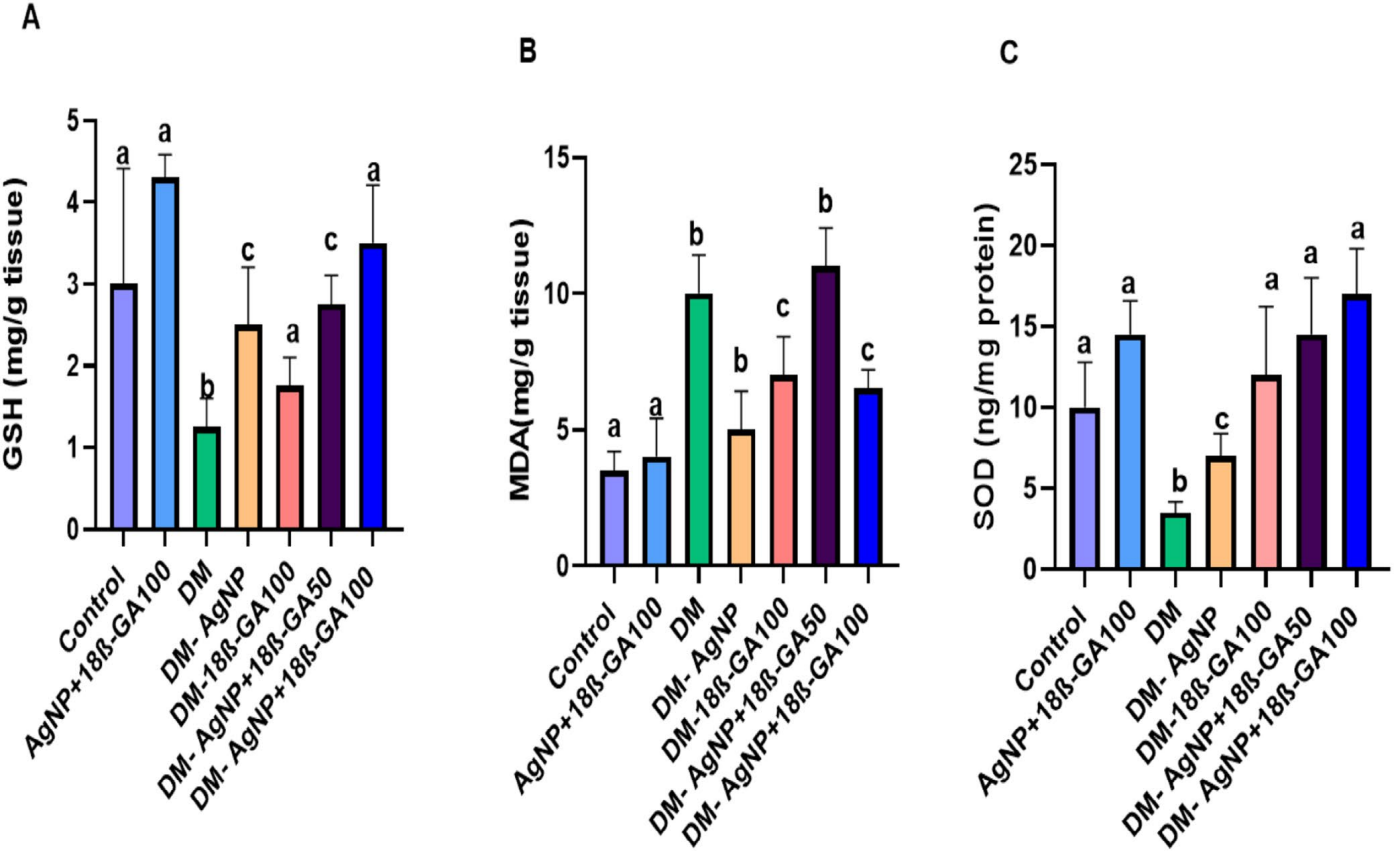
Oxidative stress parameters of testicular tissue samples obtained from experimental groups.

The results revealed that, while SOD activity and GSH levels in testicular tissue samples obtained from experimental groups decreased in the diabetes group, silver nanoparticle‐loaded 18β prevented this decrease. On the other hand, while MDA levels increased in the diabetes group, silver nanoparticle‐loaded 18β also prevented this increase (see Figure 4).

### 3.4 Serum Samples Cytokine Levels

**FIGURE 5:**
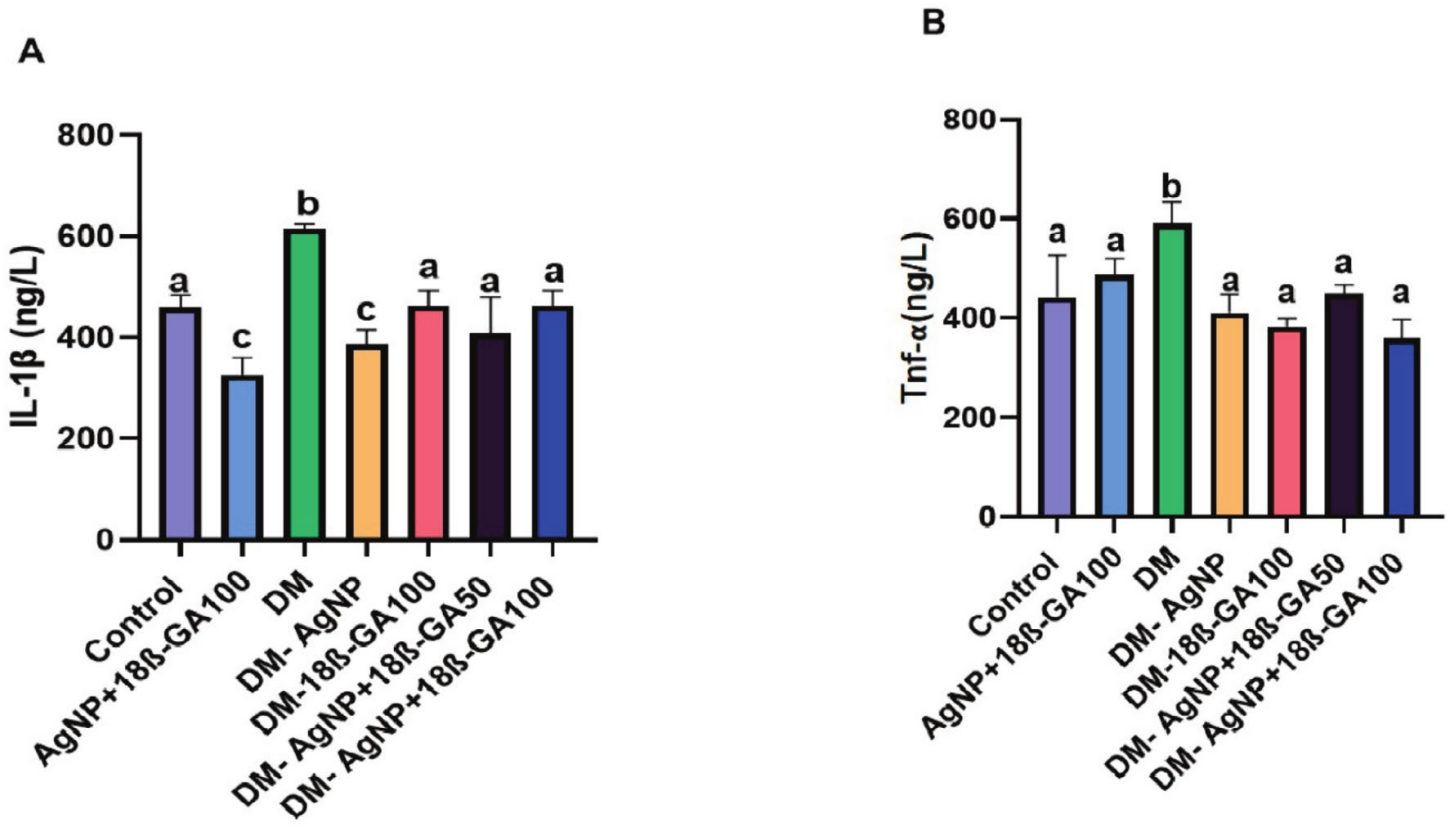
Cytokine parameters of serum samples obtained from experimental groups.

The results showed that the levels of IL‐1β and TNF‐α in the serum samples obtained from the experimental groups increased in the diabetes group, and there was a significant decrease in both the 18β group and the silver nanoparticle‐loaded 18β group as compared to the diabetes group (Figure 5).

### 3.5 Histopathological Findings

**FIGURE 6:**
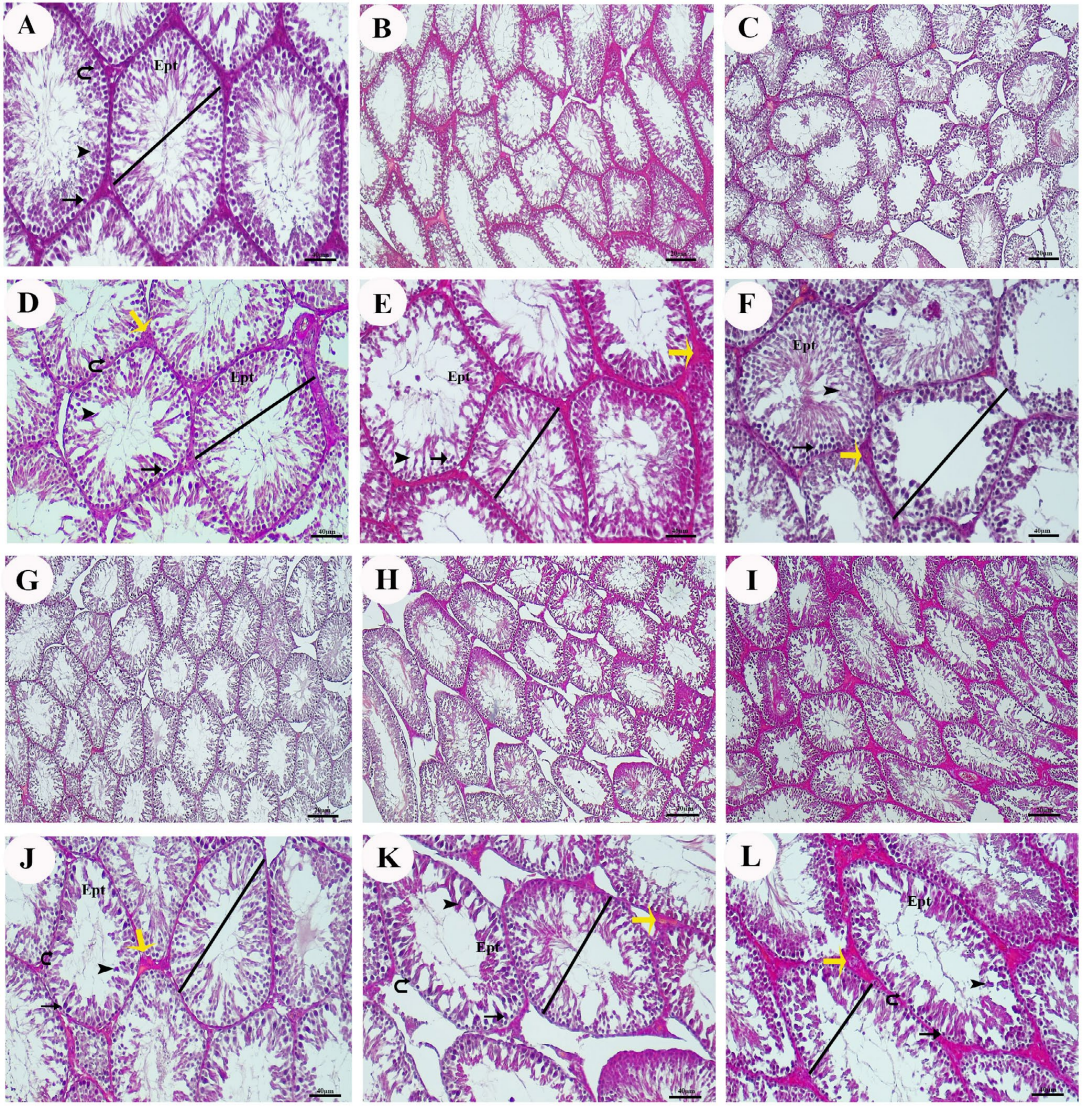
Testicular seminiferous tubules stained with Crossman's Modified Mallory's Triple Stain. Black line: Seminiferous tubule lumen and spermia, arrow: Spermatogonia, curved arrow: Sertoli cell, arrowhead: Spermatid, yellow arrow: Leydig cell, Ept: Testicular epithelium. Magnification: (B, C, G–I) 200×; (A, D–F, J–L) 400×.

A normal histological structure was observed in the testicular tissue of the control group. The tunica albuginea, which has the feature of tight connective tissue in the testes, separates the testes into lobules through septa extending inwards. We observed that the seminiferous tubules and interstitial areas exhibited normal structure within the lobules. The seminiferous tubules contained dark and light spermatogonia settled in the basement membrane, primary spermatocytes, as well as round and elongated spermatids settled toward the center of the tubules (Figure 6A–H).

A normal histological structure, which was almost identical to the one observed in the control group, was observed in the testicular tissue of the AgNP+18β‐GA100 group. We found the septa of the tunica albuginea separate the testes into lobules. The seminiferous tubules and interstitial areas exhibited normal structure. Dark and light spermatogonia, primary spermatocytes, as well as round and elongated spermatids, located toward the center of the tubules, were observed in the seminiferous tubules (see Figure 6B–I).

In the testes belonging to the diabetes (DM) group, signs of degeneration were noticeable in many seminiferous tubules. Disruptions in the spermatogenic cell series and separations at the junctions between the cells were observed. Significant dilatation was observed in the tubules. In addition, signs of separation were detected in the basal and adluminal compartments in some seminiferous tubules (see Figure 6C–J).

Signs of degeneration were also observed in the seminiferous tubules in the testis sections belonging to the diabetes (DM)‐18β‐GA100 group. Reduced damage and relatively normal seminiferous tubules were found in many tubules. The spermatogenesis process was still ongoing. In the interstitial area, decreased edema and Leydig cells with a near‐normal appearance were detected in some tubules (see Figure 6D–K).

The testes of the diabetes (DM)‐silver nanoparticle (AgNP) (DM‐AgNP)+18β‐GA50 group had degenerative changes and cellular losses in the spermatogenic cells located in the seminiferous tubule wall, intense tubular damage and degeneration, as well as significant dilatation in some tubules. Large separations in the interstitial area and a basement membrane structure separated from the tubule wall were also detected. The spermatogenesis process was considerably affected, and negative changes were also observed in the interstitial tissue (Figure 6E–L). Although tubular damage was still observed in the testicular sections of the aforementioned group, we found that tubular damage was generally reduced, and seminiferous tubules were relatively normal. The spermatogenesis process was still ongoing. In the interstitial area, decreased edema and nearly normal‐looking Leydig cells were detected in many tubules. This evidence suggests that there was some sign of recovery as compared to the sections of the diabetes group and that spermatogenesis partially continued (Figure 6F–M).

In the testicular sections of the diabetes (DM)‐silver nanoparticle (AgNP) (DM‐AgNP) group, although there was still damage in the tubules, generally decreased tubular damage and relatively normal‐structured seminiferous tubules were observed. The spermatogenesis process was ongoing. In the interstitial area, decreased edema and nearly normal‐looking Leydig cells were observed. This suggests that there were some signs of recovery as compared to the testicles of the diabetes group and that spermatogenesis partially continued (Figure 6G–N).

**FIGURE 7:**
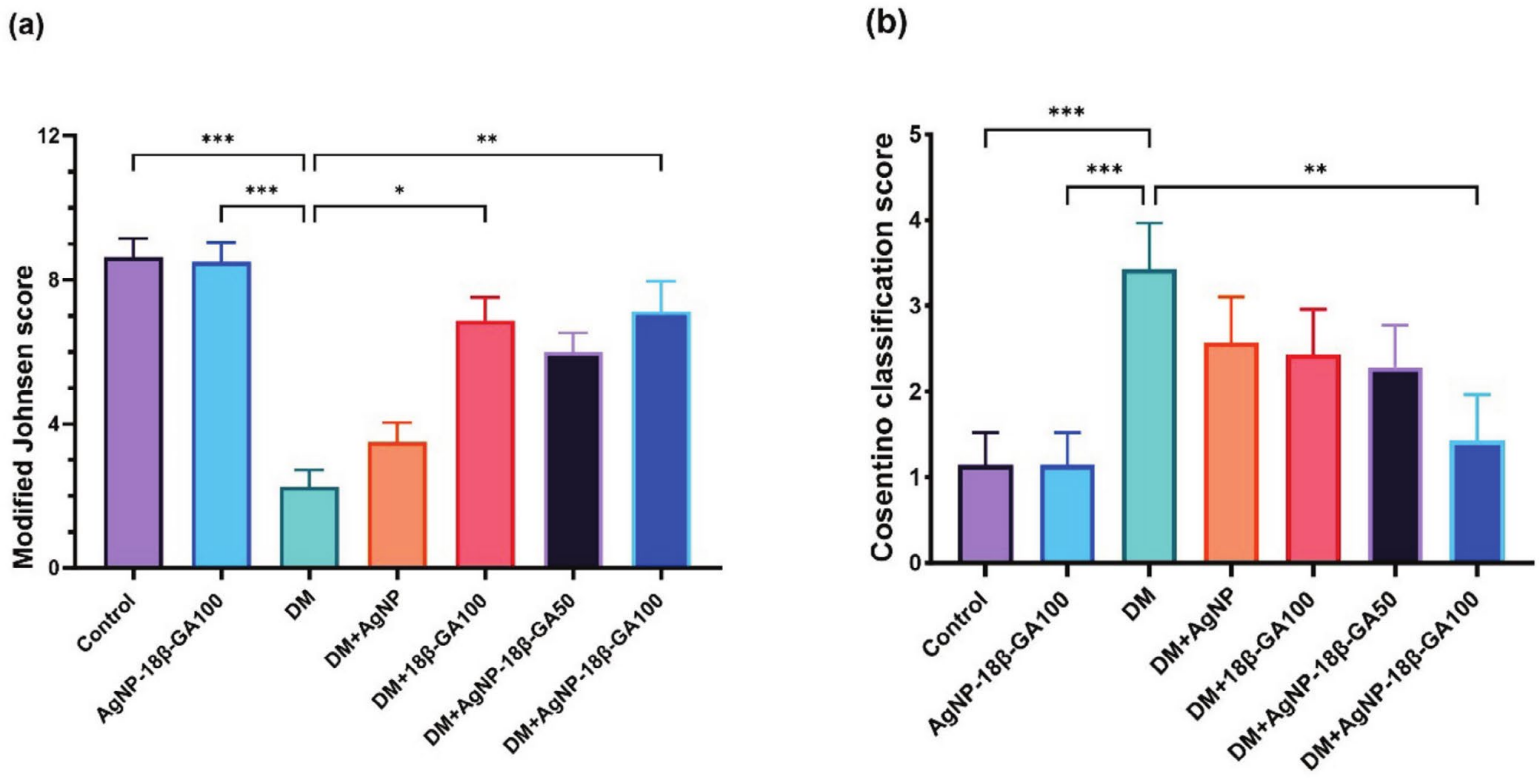
(a) Modified Johnsen scores of all groups. (b) Cosentino classification scores of all groups. The results of the entire experiment are presented as mean ± standard deviation (SD). Statistical significance: ***p < 0.001; **p < 0.01; *p < 0.05. Bars without asterisks indicate no statistically significant difference.

According to the Johnsen scoring system, the scores were 8.62 in the control group; 8.5 in the AgNP+18β‐GA100 group; 2.25 in the DM group, 6.87 in the DM‐18β‐GA100 group, 5.62 in the DM‐AgNP+18β‐GA50 group; 7.12 in the DM‐AgNP+18β‐GA100 group, and finally, 3.5 in the DM‐AgNP group (Figure 7).

### 3.6 Results of the Evaluation of Sperm Samples

At the end of the experiment, we determined that diabetes caused increases in the total testicular weight, sperm motility, density, membrane damage, abnormal sperm rate, and DNA damage in sperm samples of all experimental groups. However, the results revealed that these changes decreased in the DM+AgNPs+18β50 and DM+AgNPs+18β100 groups (see Table 4).

**TABLE 4:** Comparison of semen samples obtained from experimental groups between groups.

	Total testis weight (mg)	Motility (%)	Density (×10^6^)	Membrane damage rate (%)	Abnormal spermatozoon rate (%)	DNA damage rate
Control	4.09 ± 0.30	89.23 ± 1.18^b^	20.50 ± 1.35^d^	170.50 ± 2.17^c^	21.50 ± 1.60^b^	189.25 ± 4.06^c^
AgNP+18β‐GA100	3.05 ± 0.20	85.12 ± 1.14^b^	21.35 ± 1.15^d^	172.42 ± 1.19^c^	22.54 ± 1.20^b^	178.15 ± 3.26^c^
DM	2.65 ± 0.18	43.33 ± 4.94^a^	6.66 ± 1.22^a^	64.16 ± 3.23^a^	11.50 ± 1.11^a^	180.83 ± 3.15^c^
DM+AgNP	2.87 ± 0.06	85.00 ± 2.88^b^	18.00 ± 0.91^cd^	107.25 ± 5.57^b^	9.25 ± 1.37^a^	53.25 ± 3.98^a^ ^*^
DM+18β‐GA100	2.55 ± 0.36	80.00 ± 5.77^b^	14.25 ± 1.25^bc^	100.00 ± 3.02^b^	13.00 ± 1.68^a^	98.50 ± 5.60^b^
DM+AgNP+18β‐GA50	2.75 ± 0.14	78.75 ± 4.26^b^	10.50 ± 1.04^b^	167.25 ± 1.65^c^	24.75 ± 2.01^c^	184.75 ± 3.40^c^
DM+AgNP+18β‐GA100	3.10 ± 0.30	88.25 ± 1.18^b^	19.50 ± 1.55^d^	160.50 ± 3.37^c^	19.50 ± 1.70^b^	180.75 ± 6.06^c^

*Note:* a, b, c, d = the difference between groups in the same column.

^*^ p < 0.02 values are given as mean ± standard error.

### 3.7 Western Blot Results

**FIGURE 8:**
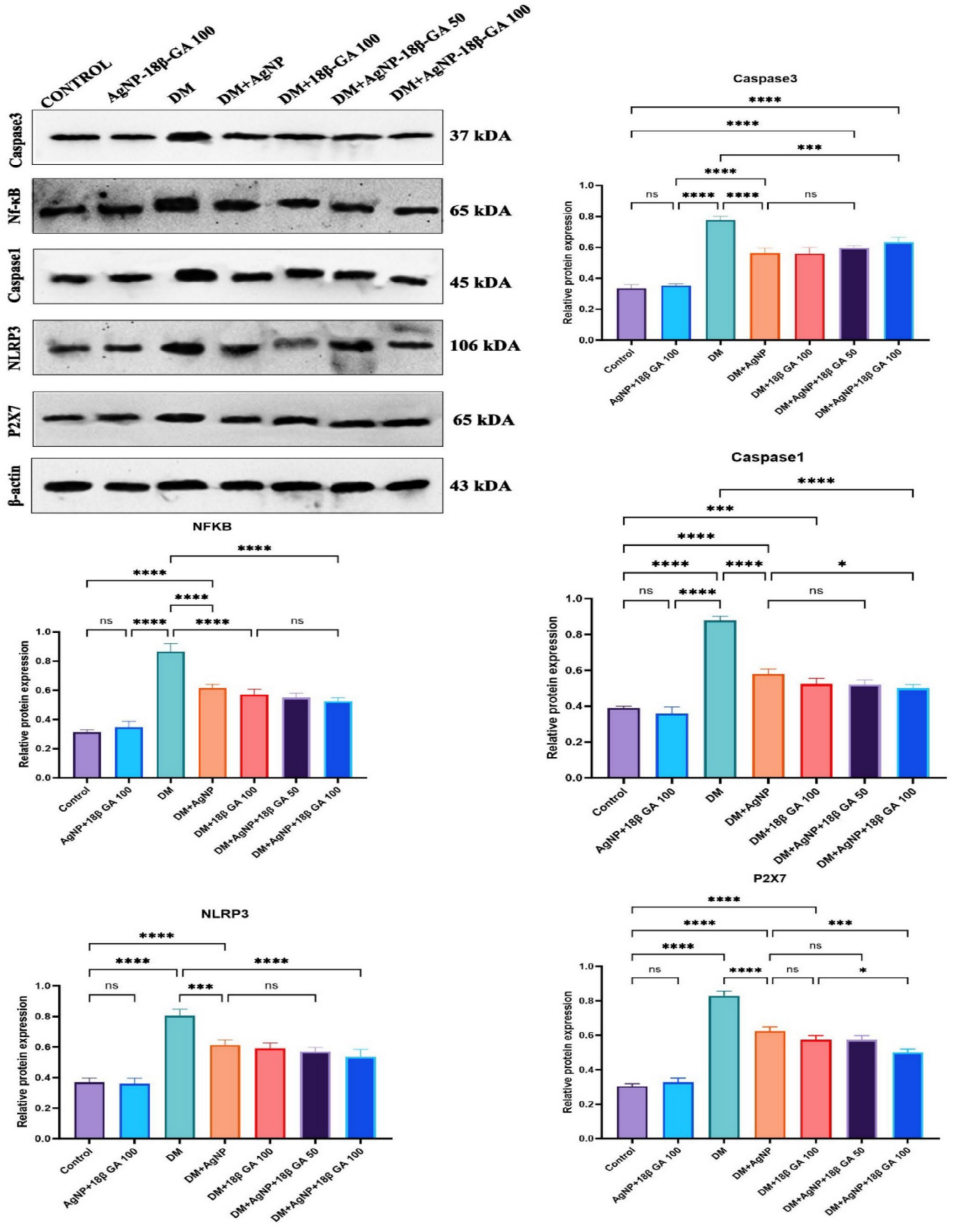
Western blot bands with relative protein expression levels of the testicular tissue of all study groups. Values are expressed as mean ± standard deviation. Asterisks (*p < 0.05, **p < 0.01, ***p < 0.001, ****p < 0.001) indicate significant differences in protein expression levels between the groups. Statistical analyses were performed using one‐way ANOVA and Tukey post hoc test, and significant differences between the tested groups are indicated.

In the evaluation of Western blot results, the results of a comparison of Caspase‐1, NLRP3, P2X7, Caspase‐3, and NF‐κB protein levels in the DM group with those in the control group showed a significant increase in the expression of all (*p* < 0.0001). The AgNP+18β‐GA 100 group turned out to be similar to the control levels, and no statistical difference was observed (*p* > 0.05). The DM+AgNP and DM+18β‐GA 100 groups showed a significant decrease in all protein markers as compared to the DM group (*p* < 0.0001). The DM+AgNP+18β‐GA 50 and DM+AgNP+18β‐GA 100 groups significantly differed from the DM group, especially in the high dose combination (*p* < 0.05). Band density values and comparisons of all groups are shown in Figure 8.

**FIGURE 9:**
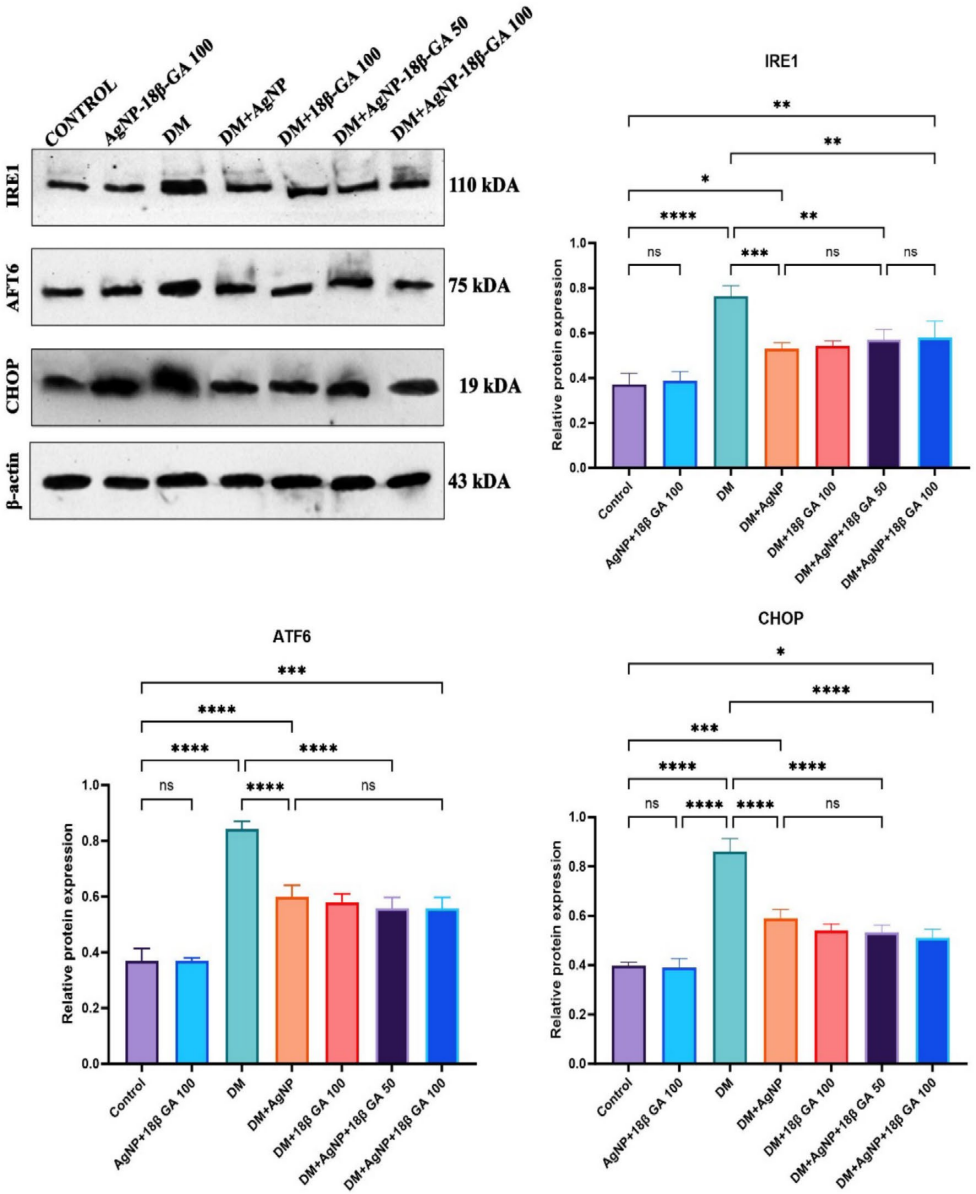
Western blot bands with relative protein expression levels of the testicular tissue of all study groups. Values are expressed as mean ± standard deviation. Asterisks (*p < 0.05, **p < 0.01, ***p < 0.001, ****p < 0.0001) indicate significant differences in protein expression levels between the groups. Statistical analyses were performed using one‐way ANOVA and the Tukey post hoc test, and significant differences between the groups are indicated.

According to the results of Western blot analysis, the DM group had significantly higher expressions of IRE1, ATF6, and CHOP proteins, which are endoplasmic reticulum stress markers, than the control group (*p* < 0.05). When only AgNP+18β‐GA 100 was applied, however, expression levels were similar to those in the control group (*p* > 0.05). However, in the comparison of the DM group with the DM+AgNP and DM+18β‐GA 100 groups, a significant decrease was observed in the expression levels of all proteins (*p* < 0.05). Finally, in the DM+AgNP+18β‐GA 50 and DM+AgNP+18β‐GA 100 groups, IRE1, ATF6, and CHOP levels significantly decreased (*p* < 0.05). Band intensities and comparisons of all groups are shown in Figure 9.

## 4 Discussion

Diabetes is a metabolic disease that occurs with the formation of high blood sugar (Shi et al. 2018, 2017). Extensive evidence indicates that diabetes is strongly associated with damage in testicular tissue. Previous research suggested that oxidative stress, inflammation, and apoptosis play a role in diabetes‐induced testicular tissue damage (Elshafey et al. 2023). To date, various treatment strategies to prevent this damage have been proposed (Koroglu Aydin et al. 2022; Zheng et al. 2024). One of these strategies is using natural agents with strong antioxidant and anti‐inflammatory effects. In the present study, we aimed to determine the effects of AgNP+18β‐GA application, which has a strong antioxidant effect, on P2X7 receptor and endoplasmic reticulum stress‐mediated NLRP3 inflammasome activation in diabetes‐induced testicular tissue.

The results revealed that Alloxan application caused diabetes in the analysis of blood glucose values of the experimental groups at 48 h, 1 week, on day 14, and at the end of the experiment. We established that blood glucose levels significantly decreased in the groups that received AgNP+18β‐GA. Similarly, in a previous study, Paul et al. (2024) determined that silver nanoparticles reduced blood glucose levels in streptozotocin‐induced diabetic rats. This suggests that, by regulating glucose metabolism, AgNP+18β‐GA treatment can limit systemic effects of diabetes and thus support improvements in testicular tissue.

Hemoglobin A1c is a very important parameter in terms of determining the level of chronic hyperglycemia (Nitin 2010). In the present study, we observed that HbA1c levels increased in the Alloxan group, while this level significantly decreased in the treatment groups. This reduction in HbA1c is particularly relevant because it reflects long‐term glycemic control, which directly correlates with the degree of microvascular and reproductive tissue damage in diabetes. Previously, Mosaad et al. reported that silver nanoparticles can alleviate the long‐term complications of hyperglycemia by reducing HbA1c levels in chronic diabetes models (Mosaad et al. 2023). Therefore, the combined decrease in fasting glucose and HbA1c levels in our study suggests that AgNP+18β‐GA not only exerts acute metabolic regulation but also mitigates chronic hyperglycemic stress on the reproductive system.

In the present study, the significant decrease in SOD activity and GSH levels, along with the marked elevation in MDA levels in the diabetic group, confirms the presence of oxidative stress–mediated testicular damage in diabetes. Similar findings have been reported in previous studies, where hyperglycemia induced excessive reactive oxygen species (ROS) production, leading to the depletion of enzymatic (SOD, CAT, GPx) and non‐enzymatic (GSH) antioxidants, while accelerating lipid peroxidation as indicated by increased MDA concentrations (Elshafey et al. 2023; Koroglu Aydin et al. 2022). In agreement with our results, importantly, in our study, treatment with AgNP+18β‐GA effectively prevented the decline in SOD and GSH, while suppressing MDA accumulation. This protective profile is comparable to that reported for various antioxidant agents, such as quercetin (Tvrdá et al. 2022) and curcumin (Kanter et al. 2013), which have been shown to restore redox balance and mitigate oxidative injury in diabetic reproductive tissues. Previous studies have determined that 18β‐GA is a powerful antioxidant and prevents oxidative stress‐induced pleasure in various tissues (Darendelioglu et al. 2025; Caglayan et al. 2022). Moreover, the enhanced efficacy observed with silver nanoparticle‐mediated delivery supports previous evidence that nanocarrier systems can improve the bioavailability, stability, and tissue penetration of bioactive compounds, thereby potentiating their antioxidant action (Mukherjee et al. 2024). Collectively, our findings indicate that AgNP+18β‐GA offers an effective strategy to counteract diabetes‐induced oxidative stress in the testes, with outcomes consistent with and, in some aspects, superior to conventional antioxidant interventions.

TNF‐α, IL‐1β, and NF‐κB are important parameters that indicate the onset of the inflammatory process in the cell (Sengul et al. 2023; Gelen et al. 2024; Yakut et al. 2024). Conversely, available evidence indicates that stimulation of the P2X7R receptor stimulates inflammation via extracellular ATP, and subsequently, the formation of the NLRP3 inflammasome complex and subsequent caspase‐1 activation and IL‐1β production occur (Pelegrin 2021). In our study, these parameters, which play an important role in the inflammatory process, significantly increased in the diabetes group. Elevated TNF‐α and IL‐1β levels indicate an activated pro‐inflammatory milieu, while NF‐κB upregulation reflects transcriptional activation of multiple inflammation‐related genes. These inflammatory changes can directly impair spermatogenesis and compromise sperm quality. In line with these findings, various previous studies showed that diabetes triggers the inflammatory process by stimulating P2X7 release in testicular tissue (Qian et al. 2023). For instance, Ding et al. showed that diabetes increases NLRP3 expression (Ding et al. 2019). In another relevant study, Kong et al. demonstrated the effectiveness of the P2X7/NLRP3 signaling pathway in diabetes‐induced retinopathy (Kong et al. 2022). Similarly effective was flavonoid application on the P2X7/NLRP3 signaling pathway (Akcay and Karatas 2024). In yet another previous study, Ye et al. (2022) identified the positive effect of the NLRP3 signaling pathway in diabetes‐mediated atherosclerosis. In our results, we observed that AgNP+18β‐GA prevented the activation of the P2X7 receptor and the NLRP3 inflammasome, thereby improving the damage in the testicular tissue. This dual suppression of upstream (P2X7R) and downstream (NLRP3 inflammasome) inflammatory triggers likely reduces caspase‐1 activation and IL‐1β maturation, thus minimizing inflammatory injury to germ cells.

ER is an organelle highly sensitive to intracellular and extracellular stimuli (Flessa et al. 2022). When ER stress is activated for any reason, the amount of unfolded or misfolded proteins in the cell increases, and, accordingly, three transmembrane proteins called p‐PERK, IRE1α, and ATF6, which bind unfolded proteins, are separated from GRP78 (Kara et al. 2023). In experimental settings, ER stress can be induced in various tissues with diabetes in rats (Eizirik et al. 2008); when ER stress occurs, the expression levels of GRP78, CHOP, ATF6, p‐IRE1, sXBP1, and p‐PERK increase. In our results, CHOP, ATF6, and p‐IRE1 expression increased in diabetes‐induced testicular tissue. Elevated CHOP expression is particularly important as it is a pro‐apoptotic marker, suggesting that ER stress may contribute to germ cell loss in diabetes. Similarly, ATF6 and p‐IRE1 upregulation reflect activation of the unfolded protein response, which, when prolonged, can exacerbate cell injury. Several previous studies reported that 18β‐GA application suppresses endoplasmic reticulum stress (Caglayan et al. 2022; Shen et al. 2017). In our study, we found that AgNP+18β‐GA decreased the expression of CHOP, ATF6, and p‐IRE1 in testicular tissue in rats. This downregulation indicates that the treatment helps restore ER homeostasis, preventing apoptosis and maintaining normal spermatogenic cell function.

Caspase‐1, NLRP3, P2X7, Caspase‐3, and NF‐κB are key molecular players involved in inflammation and cell death pathways. The NLRP3 inflammasome, a multiprotein complex, is activated in response to various cellular stress signals, often triggered by extracellular ATP binding to the P2X7 receptor. This activation promotes the recruitment and activation of Caspase‐1, which subsequently processes pro‐inflammatory cytokines such as IL‐1β and IL‐18 into their mature forms, amplifying the inflammatory response. NF‐κB, a pivotal transcription factor, regulates the expression of inflammasome components and pro‐inflammatory mediators, thus acting upstream in the priming phase of NLRP3 activation. In parallel, Caspase‐3, a key executioner protease in apoptosis, may be activated during inflammasome‐mediated cell death (pyroptosis) or through other apoptotic signaling cascades, contributing to tissue injury and disease progression. Together, these molecules form a tightly regulated network that links inflammation, immune signaling, and programmed cell death (El Helew et al. 2024; Eriten et al. 2024; Caglayan et al. 2025).

Our examination of testicular tissue and sperm parameters showed decreased testicular weight and suppressed spermatogenesis in diabetic rats. Reduced motility, lower sperm density, and higher membrane damage rates indicated marked deterioration in sperm quality. The increased proportion of abnormal spermatozoa and elevated DNA damage rates suggested that diabetes causes both functional and genetic impairments. These findings align with previous studies reporting alterations in sperm morphology, DNA fragmentation, and motility loss in experimental diabetes models (Kızıl Emre et al. 2023)

Treatment with AgNP+18β‐GA significantly improved testicular weight, sperm motility and density, and reduced membrane damage, morphological abnormalities, and DNA damage. These results suggest that the treatment restores spermatogenesis by suppressing oxidative and inflammatory stress in the testicular microenvironment. The reduction in DNA damage may also be linked to decreased inflammasome activity and ER stress. Our findings are consistent with Erbaş et al., who reported that oleuropein‐loaded AgNPs improved sperm parameters and protected testicular tissue (Erbaş et al. 2024)

Histopathological evaluation revealed degeneration, necrosis, and structural deterioration in the seminiferous tubules of diabetic rats, consistent with Liu et al.'s observations (Liu et al. 2019). Several studies have also shown that flavonoids can protect against diabetes‐induced testicular injury (Zheng et al. 2024). In line with this evidence, AgNP+18β‐GA treatment in our study restored the seminiferous tubules, reorganized germ cells, and reduced tubular sclerosis. This agrees with Khan et al., who demonstrated that silver nanoparticles ameliorated histological damage in diabetic rats (Khan et al. 2023)

Overall, our results indicate that AgNP+18β‐GA alleviates diabetes‐induced testicular damage by reducing inflammation and oxidative stress, protecting both cellular structure and function. The treatment appears to suppress P2X7 receptor activity, downregulate the NLRP3 inflammasome, modulate ER stress responses, and restore redox balance. These combined effects likely contribute to improved sperm parameters and may enhance fertility potential in diabetic conditions

## Author Contributions

Volkan Gelen, Adem Kara, Ali Yeşildağ, Deniz Tekiner, Hülya Kara, and Serkan Ali Akarsu designed and performed the research, analyzed data, and edited the article.

## Ethics Statement

The study was approved by Kafkas University Animal Experiments Local Ethics Committee (KAÜ‐HADYEK/2023‐036). All experiments were performed in accordance with the European Directive 2010/63/EU for animal experiments. In addition, all animal‐related procedures used in this study were performed in accordance with Animal Research: Reporting of In Vivo Experiments (ARRIVE) guidelines.

## Conflicts of Interest

The authors declare no conflicts of interest.

## Data Availability

No additional data are available.
